# Indole-3-Carboxaldehyde Restores Gut Mucosal Integrity and Protects from Liver Fibrosis in Murine Sclerosing Cholangitis

**DOI:** 10.3390/cells10071622

**Published:** 2021-06-29

**Authors:** Fiorella D’Onofrio, Giorgia Renga, Matteo Puccetti, Marilena Pariano, Marina Maria Bellet, Ilaria Santarelli, Claudia Stincardini, Paolo Mosci, Maurizio Ricci, Stefano Giovagnoli, Claudio Costantini, Luigina Romani

**Affiliations:** 1 Department of Medicine and Surgery, University of Perugia, Piazzale Lucio Severi 1, 06132 Perugia, Italy; donofrio.fiorella@libero.it (F.D.); rengagiorgia@gmail.com (G.R.); marilena.pariano@gmail.com (M.P.); marinamaria.bellet@unipg.it (M.M.B.); ilasanta@libero.it (I.S.); claudiastincardini@gmail.com (C.S.); paolo.mosci@unipg.it (P.M.); costacla76@gmail.com (C.C.); 2 Department of Pharmaceutical Sciences, University of Perugia, Via del Liceo 1, 06123 Perugia, Italy; matteo.puccetti@gmail.com (M.P.); maurizio.ricci@unipg.it (M.R.); stefano.giovagnoli@unipg.it (S.G.); 3 University Research Center on Functional Genomics (C.U.R.Ge.F), University of Perugia, 06132 Perugia, Italy

**Keywords:** primary sclerosing cholangitis, aryl hydrocarbon receptor, indole-3-carboxaldehyde, mucosal barrier, microbiota

## Abstract

Primary sclerosing cholangitis (PSC) is a long-term liver disease characterized by a progressive course of cholestasis with liver inflammation and fibrosis. Intestinal barrier dysfunction has been implicated in the pathogenesis of PSC. According to the “leaky gut” hypothesis, gut inflammation alters the permeability of the intestinal mucosa, with the translocation of gut-derived products that enter the enterohepatic circulation and cause hepatic inflammation. Thus, the administration of molecules that preserve epithelial barrier integrity would represent a promising therapeutic strategy. Indole-3-carboxaldehyde (3-IAld) is a microbial-derived product working at the interface between the host and the microbiota and is able to promote mucosal immune homeostasis in a variety of preclinical settings. Herein, by resorting to a murine model of PSC, we found that 3-IAld formulated for localized delivery in the gut alleviates hepatic inflammation and fibrosis by modulating the intestinal microbiota and activating the aryl hydrocarbon receptor-IL-22 axis to restore mucosal integrity. This study points to the therapeutic potential of 3-IAld in liver pathology.

## 1. Introduction

Primary sclerosing cholangitis (PSC) is a rare, slowly progressing and chronic liver disease characterized by inflammation and the destruction of the biliary tree that leads to fibrosis or end-stage complications, such as liver cirrhosis [1]. The development of PSC may be preceded by a silent asymptomatic period, finally resulting in fatigue, jaundice, and pruritus, and leading to stricturing, cholestasis, and progressive liver dysfunction [2]. To date, the pathogenesis of human PSC is still unclear, but available evidence points to a multifactorial etiology, influenced by genetic predisposition and immune-mediated processes [2,3,4].

PSC exhibits remarkable associations with inflammatory bowel disease (IBD) and enteric microbial dysbiosis. Gut microbiota is normally confined in the intestinal lumen and engages in reciprocal beneficial activities with the host during homeostasis. A functional imbalance in the microbial ecosystem favors epithelial barrier dysfunction and increased intestinal permeability, leading to the translocation of gut-derived products into the portal venous system, with consequent biliary inflammation and liver fibrosis [5,6]. Thus, the “gut-liver axis” has been implicated in the pathogenesis of liver diseases, such as PSC [7,8,9]. In the last few years, several studies have tried to deeply characterize the clinical PSC–IBD phenotype, providing insight into PSC pathogenesis that could be exploited to develop innovative therapies.

Despite many scientific advances made over recent years, PSC remains a substantial clinical challenge. To date, no effective medical therapy has significantly improved clinical outcomes, and most patients ultimately require liver transplantation. Therefore, PSC is still an orphan disease. The current medical management for PSC includes the use of molecules to regulate the bile acid metabolism, immune-modulators, and anti-inflammatory and anti-fibrotic drugs [2,10,11]. Even though the use of some treatments (i.e., ursodeoxycholic acid) can improve symptoms, they fail to block the progression of cholestatic liver disease, prompting the development of alternative therapeutic strategies.

We have previously demonstrated that indole-3-carboxaldehyde (3-IAld), abundantly produced by the commensal *Lactobacillus reuteri* in condition of unrestricted tryptophan availability, acts as a ligand of the aryl hydrocarbon receptor (AhR) [12]. Evidence has since accumulated demonstrating the beneficial activity of 3-IAld in a variety of preclinical settings, which includes the restoration of intestinal barrier integrity via the AhR/IL-22 axis [13]. In particular, 3-IAld formulated for intestinal delivery restored gut and liver function and prevented the metabolic complications associated with the intake of a high-fat diet [14,15,16]. Despite being controversial [17], the AhR-IL-22 axis is also thought to be involved in liver homeostasis in mice by promoting liver regeneration [18] and preventing fibrogenesis [19,20,21].

In the current study, we resorted to a murine model of PSC based on the administration of 3,5-diethoxycarbonyl-1,4-dihydrocollidine (DDC) and evaluated the effect of orally delivered 3-IAld-loaded enteric microparticles (3-IAld-MP) [22] in the protection against liver pathology. We found that 3-IAld-MP administration prevented liver inflammation and fibrosis through the maintenance of intestinal mucosal immune homeostasis. Thus, by acting through the “gut-liver axis”, 3-IAld may preserve liver function.

## 2. Materials and Methods

### 2.1. Mice

C57BL/6 mice were purchased from Charles River Laboratories (Calco, Italy). *Il22^–/–^* mice were bred under specific pathogen-free conditions in the Animal Facility of Perugia (Perugia, Italy). The 6–8-week-old male and female mice, weighing 20 to 25 g, were used in all experiments. Murine experiments were performed according to the Italian Approved Animal Welfare Authorization 662/2020-PR (valid for two years (2020–2022)) and Legislative decree 26/2014, regarding the animal license obtained by the Italian Ministry of Health. 

### 2.2. Mouse Model of DDC-Induced Sclerosing Cholangitis

For the murine sclerosing cholangitis model, the mice were given a 0.1% DDC-supplemented diet (Mucedola Srl, Milan, Italy) for 14 days (active phase). After the DDC diet, the mice were given normal chow and drinking water for the successive 14 days (recovery phase) [23]. The mice were randomly divided into 3 groups. Each group was housed in the same cage, fed and treated as follows: (a) the vehicle group was fed a control chow diet with oral gavage of distilled water, (b) the 0.1% DDC group, (c) the 0.1% DDC group treated with 18 mg/kg of 3-IAld every other day. The mice were euthanized at different time points. 

### 2.3. Enteric Formulation Preparation

Briefly, the enteric microparticles (MP) were prepared using Eudragit^®^ L100 to S100 (Rohm Pharma GmbH, Darmstadt, Germany) at a ratio of 1:2, with the addition EC (30% *w*/*w*, ETHOCEL std. 7, Dow Chemical Company, Milan, Italy), as described in [22]. 3-IAld (Sigma-Aldrich, Milan, Italy) and the polymers were dissolved in ethanol at a feedstock concentration of 3% *w/v*, and spray-dried at an inlet temperature of 75 °C, using a Mini Spray-dryer model B-290 (Büchi, Milan, Italy) in the co-current mode, equipped with a two-fluid nozzle with a 0.7 mm nozzle tip and a 1.5 mm diameter nozzle cap. The aspirator capacity was maintained at 20 m^3^/h, the airflow rate was 301 L/h, and the feed rate 2.4 mL/min. The obtained dried MP were recovered by using a high-performance cyclone (Büchi, Milan, Italy).

### 2.4. Histology 

The tissues were removed and fixed in 10% phosphate-buffered formalin (Bio Optica, Milan, Italy), embedded in paraffin, and sectioned at 3 μm. For the histology, the paraffin-embedded tissues were stained with Hematoxylin and Eosin (H&E), Periodic Acid–Schiff (PAS), and Masson’s trichrome staining. For the quantification of porphyrin deposition and fibrosis, the data were analyzed using ImageJ software (US National Institutes of Health, Bethesda, MD, USA) in randomly chosen fields, with 20× and 40× objectives. Based on the existing literature [24], for the colon histological score, 4 components were assessed: “inflammation extent”, “damage in crypt architecture”, “hyperemia/edema” and “grade of accumulation with inflammatory cells”. The colonic sections were scored with 0 to 3 points for each parameter. The total histological score, ranging from 0 to 12, was obtained by summing the 4 histological components’ scores.

### 2.5. Immunohistochemistry 

For immunohistochemistry staining, the liver sections were rehydrated and the antigens were retrieved by boiling in a citrate buffer (10 mM, pH 6). Subsequently, the endogenous peroxidase was quenched with 3% H_2_O_2_ for 10 minutes at room temperature and then incubated with a blocking buffer (10% Horse Serum in TBS). After rinsing, the slides were treated overnight at 4 °C with primary antibodies α-SMA and CD11b (Invitrogen, Thermo Fisher Scientific, Waltham, MA, USA) and F4/80 (Santa Cruz Biotechnology, Dallas, TX, USA). The slides were then incubated with biotinylated anti-rat IgG or biotinylated anti-mouse IgG (Fisher Scientific, Thermo Fisher Scientific). Antibody binding was detected with a VECTASTAIN Elite ABC Kit (VECTOR Laboratories, Maravai LifeSciences, Burlingame, CA, USA). Diaminobenzidine was used as a chromogen, followed by counterstaining with hematoxylin. Images were acquired using a microscope BX51 microscope (Olympus, Shinjuku, Tokyo, Japan) and captured using a high-resolution DP71 camera (Olympus).

### 2.6. Immunofluorescence

For immunofluorescence, the colon sections were rehydrated and, after antigen retrieval in a citrate buffer (10 mM, pH 6), fixed in 4% formaldehyde (ChemCruz, TE Huissen, The Netherlands) for 20 min at room temperature and permeabilized in a blocking buffer containing 3% BSA. The slides were then incubated at 4 °C with primary antibodies anti-ZO1 (Invitrogen) and anti-Ki67 (Abcam, Cambridge, UK). After extensive washing with PBS, the slides were then incubated at room temperature for 60 min with secondary antibodies goat anti-Rabbit 550 (Bethyl Laboratories, Montgomery, TX, USA) and goat anti-Rabbit 488 (Invitrogen). The nuclei were counterstained with Hoechst 33342 (Invitrogen). Images were acquired using a microscope BX51 microscope (Olympus) and captured using a high-resolution DP71 camera (Olympus).

### 2.7. TUNEL Assay

Terminal deoxynucleotidyl transferase dUTP nick-end-labeling (TUNEL) staining was performed using the In Situ Cell Death Detection Kit, POD (Roche Diagnostics, Mannheim, Germany) according to the manufacturer’s instructions. The sections were mounted and analyzed by fluorescent microscopy, and TUNEL-positive cells were quantified as the percentage of positive cells from 4 high-powered fields in each section [25].

### 2.8. Serum Biochemical Analyses

Blood samples were centrifuged at 2.000 g for 10 minutes and stored at −80 °C until the analyses could be performed. Serum levels of total bilirubin, alkaline phosphatase, and alanine aminotransferase were analyzed by using specific kits (Elabscience, Houston, TX, USA and BioVison, Milpitas, CA, USA).

### 2.9. ELISA

Cytokine and sCD14 contents were determined in the liver homogenates and serum by using specific ELISA kits according to the manufacturers’ instructions (Biolegend, San Diego, CA, USA; eBioscience Inc. and Life Technologies, Thermo Fisher Scientific; and R&D System, Minneapolis, MN, USA). The concentration of secreted cytokines in the supernatants was normalized to the total tissue protein and expressed as the picogram of cytokine per microgram of total protein.

### 2.10. Bacterial DNA Extraction 

Fecal bacterial DNA was extracted using a QIAamp Fast DNA Stool Mini Kit (Qiagen, Hilden, Germany) following the manufacturer’s instructions. Bacteria species-specific PCR was carried out with primers targeted on the 16S rRNA, using the a CFX96 Touch Real-Time PCR detection system and an iTaq Universal SYBR Green Supermix (Bio-Rad, Hercules, CA, USA). Bacterial abundances were expressed as relative 16S rRNA gene levels. The following bacterial primers were used: Eubacteria (forward: ACTCCTACGGGAGGCAGCAG, reverse: ATTACCGCGGCTGCTGG); Actinobacteria (forward: TACGGCCGCAAGGCTA, reverse: CGCGGCCTATCAGCTTGTTG); Bacteroidetes (forward: GGARCATGTGGTTTAATTCGATGAT, reverse: AGCTGACGACAACCATGCAG); *E. coli* (forward: CAAGTCATCATGGCCCTTAC, reverse: CGGACTACGACGCACTTTAT); Firmicutes (forward: GGAGYATGTGGTTTAATTCGAAGCA, reverse: AGCTGACGACAACCATGCAC); *L. reuteri* (forward: ACCGAGAACCACCGCGTTATTT, reverse: CATAACTTAACCTAAACAATCAAAGATTGTCT); Proteobacteria (forward: CCGCAAGGTTAAAACTCAAAGGAA, reverse: CAGACATGTCAAGGGTAGGTAAGG).

### 2.11. RT-PCR

Real-time PCR was performed using the CFX96 Touch Real-Time PCR detection system and an iTaq Universal SYBR Green Supermix (Bio-Rad). The livers were lysed and the total RNAs were isolated with TRIZOL Reagent (Thermo Fisher Scientific), and the cDNAs were synthesized using a PrimeScript RT Reagent Kit with a gDNA Eraser (Takara, Kusatsu, Japan), according to the manufacturers’ instructions. The amplification efficiencies were validated and normalized against β-actin. Each data point was examined for integrity by analysis of the amplification plot. The thermal profile for the SYBR Green RT-PCR was at 95 °C for 3 min, followed by 40 cycles of denaturation for 30 s at 95 °C, and an annealing/extension step of 30 s at 60 °C. Each data point was examined for integrity by analysis of the amplification plot. The following murine primers were used: *β-actin* (forward: AGCCATGTACGTAGCCATCC, reverse: CTCTCAGCTGTGGTGGTGAA); *AhR* (forward TCCATCCTGGAAATTCGAACC, reverse TCTTCATCCGTCAGTGGTCTC; *Bax*: forward: GATGAACTGGACAGCAATATGG, reverse: CGGAAGAAGACCTCTCGG); *Bcl2* (forward: ACGAGTGGGATGCTGGAGATG, reverse: TCAGGCTGGAAGGAGAAGATGC); *Cyp1a1* (forward: ACAGTGATTGGCAGAGATCG, reverse: GAAGGGGACGAAGGATGAAT); *Tgfb1* (forward: ATATTTGGAGCCTGGACACA, reverse: CGTAGTAGACGATGGGCAGT).

### 2.12. Statistical Analysis

The data are expressed as the mean ± SEM. Statistical significance was calculated by using one-way ANOVA with the Bonferroni post-hoc test correction for multiple comparisons and by the two-tailed Student’s t-test for single comparisons. The histological scores were compared using the Kruskal–Wallis test. The data reported are pooled or representative of 3 experiments. The in vivo groups consisted of 4 mice/group. GraphPad Prism software 6.01 (GraphPad Software, San Diego, CA, USA) was used for analysis.

## 3. Results 

### 3.1. Evaluation of DDC Diet-Induced Liver Injury

**Figure 1:**
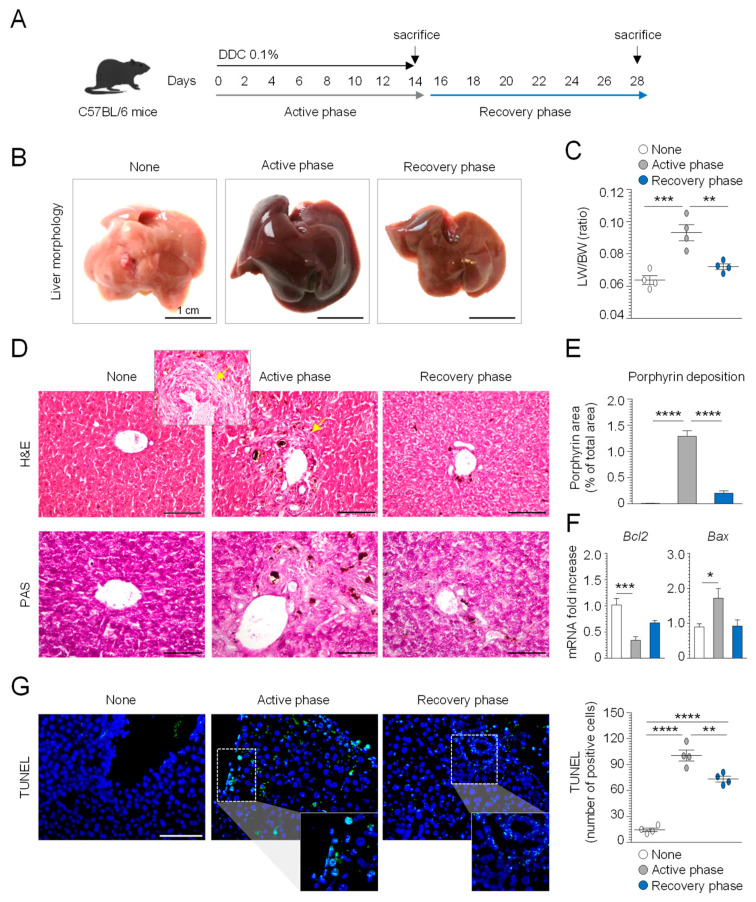
Characterization of the sclerosing cholangitis model in C57BL/6 mice. C57BL/6 mice were fed for 14 days with a special diet containing 0.1% xenobiotic 3,5-diethoxycarbonyl-1,4-dihydrocollidine (DDC) (active phase) and subjected to a recovery time of 14 days (recovery phase), as depicted in the experimental schedule (**A**). The mice were sacrificed at different time points and evaluated for (**B**) liver gross histology, (**C**) liver (LW) and body weight (BW) ratio, (**D**) liver histopathology by Hematoxylin and Eosin (H&E), and Periodic Acid–Schiff (PAS) stains, (**E**) porphyrin deposition, (**F**) apoptotic marker expression by RT-PCR, and (**G**) TUNEL staining in the liver. Hoechst was used for nuclear counterstain in blue. Photographs were taken with a high-resolution microscope (Olympus BX51), 20× magnification (scale bars, 200 μm). The data are presented as the mean ± SEM and are representative of three experiments (*n* = 4 mice/group). * *p* < 0.05, ** *p* < 0.01, *** *p* < 0.001, **** *p* < 0.0001, DDC-treated vs. untreated (none) mice, and active phase vs recovery phase. One-way ANOVA, Bonferroni post-test.

DDC feeding in mice is a well-established model that reproduces the main histopathological hallmarks of human PSC, such as liver damage and inflammation, occurring in the early stage of disease, and periductular fibrosis, which characterizes the end stage of liver disease [26]. DDC withdrawal is followed by a partial reversal of cholestasis, thus allowing the exploration of the different phases of murine sclerosing cholangitis [27]. In order to explore the potential therapeutic application of 3-IAld-MP in the prevention and/or reversal of liver damage, we first characterized the C57BL/6 mice fed a 0.1% DDC-supplemented diet for 14 days (active phase), followed by 14 days of a normal diet (recovery phase), as depicted in Figure 1A. Liver macroscopic analysis showed a dark-brownish color change and hepatomegaly in the active phase (Figure 1B), with a significant increase in the liver-to-body weight ratio (Figure 1C). Of interest, the glycogen storage, revealed by PAS-staining, was significantly reduced in the active phase (Figure 1D). Additionally, the H&E staining of the liver sections showed porphyrin plugs in the bile ductules, and a small number of “onion skin”-like fibrotic rings around the larger ducts (Figure 1D,E), which is a typical histological feature of human PSC. In contrast, mice subjected to a recovery period showed a substantial amelioration of liver tissue pathology with a significant decrease in porphyrin deposition and an increase in glycogen storage (Figure 1B–E). To quantify the impact of DDC feeding on cellular damage, we assessed the cellular apoptosis in the active and recovery phases. We observed increased cellular apoptosis, as revealed by the TUNEL assay (Figure 1G) and the expression of the apoptotic markers Bcl2 and Bax (Figure 1F) in the early stage of DDC-induced sclerosing cholangitis, which was reversed after the recovery phase. These data confirm that DDC administration induces cholestatic liver damage and that the cessation of DDC intoxication can promote hepatocytes regeneration in the recovery phase.

### 3.2. 3-IAld Reduces Inflammation and Alleviates Hepatic Fibrosis

**Figure 2:**
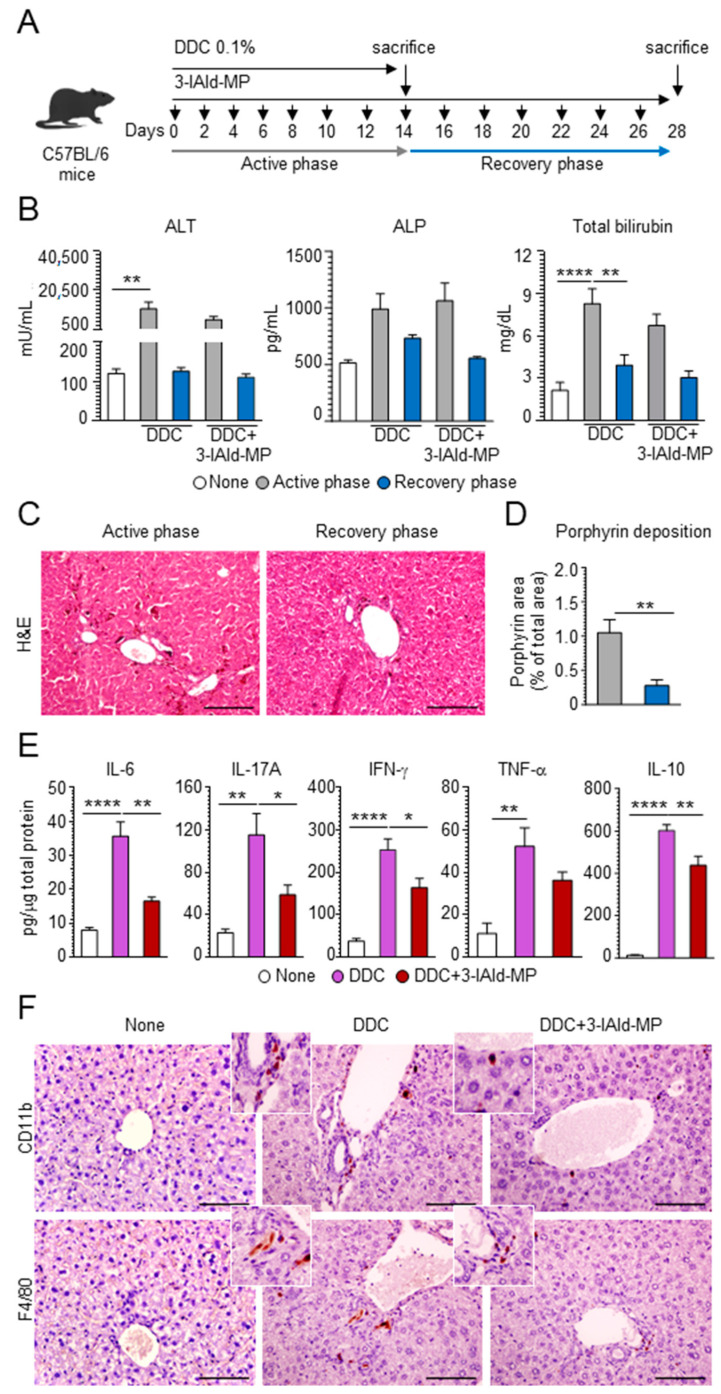
3-IAld-MP reduces liver inflammation. C57BL/6 mice were fed for 14 days with a 0.1% DDC-containing diet (active phase) and subjected to a recovery time of 14 days (recovery phase). The mice were treated intragastrically with 3-IAld-MP every other day throughout the experiment, as depicted in the experimental schedule (**A**). The mice were evaluated for (**B**) serum levels of alanine aminotransferase (ALT), alkaline phosphatase (ALP), and total bilirubin, (**C**) liver histopathology by H&E, (**D**) porphyrin deposition, (**E**) cytokine production by ELISA, and (**F**) liver CD11b^+^ and F4/80^+^ cells by immunohistochemistry. Photographs were taken with a high-resolution microscope (Olympus BX51), 40× magnification (scale bars, 100 μm). The data are presented as the mean ± SEM and are representative of three experiments (*n* = 4 mice/group). * *p* < 0.05, ** *p* < 0.01, **** *p* < 0.0001, DDC-treated vs untreated (none) mice, and DDC vs. DDC + 3-IAld-MP. One-way ANOVA, Bonferroni post-test, and two-tailed Student’s t-test.

**Figure 3:**
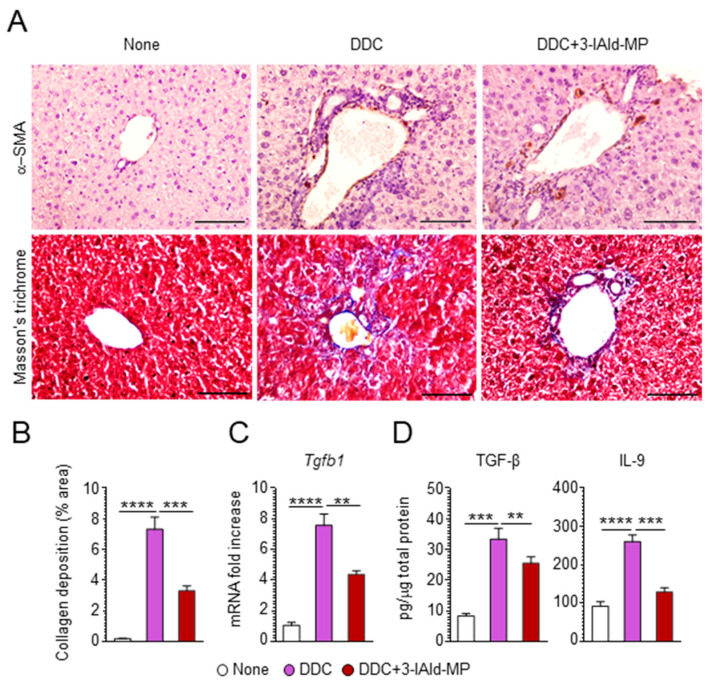
3-IAld-MP alleviates liver fibrosis. C57BL/6 mice were fed for 14 days with a 0.1% DDC-containing diet and subjected to a recovery time of 14 days. The mice were treated intragastrically with 3-IAld-MP every other day throughout the experiment, as depicted in Figure 2. The mice were evaluated for (**A**) fibroblast activation (immunohistochemistry for α-SMA), collagen deposition (Masson’s trichrome staining) and (**B**) quantification, in liver sections, (**C**) *Tgfb1* expression by RT-PCR, and (**D**) TGF-β and IL-9 levels by ELISA in liver homogenates. Photographs were taken with a high-resolution microscope (Olympus BX51), 40× magnification (scale bars, 100 μm). The data are presented as the mean ± SEM and are representative of three experiments (*n* = 4 mice/group). ** *p* < 0.01, *** *p* < 0.001, **** *p* < 0.0001, DDC-treated vs untreated (none) mice, and DDC vs. DDC + 3-IAld-MP. One-way ANOVA, Bonferroni post-test.

To assess whether 3-IAld would prevent DDC-induced sclerosing cholangitis, we treated DDC-fed mice with 3-IAld-MP intragastrically at the beginning of treatment with DDC, as depicted in Figure 2A. We first evaluated the effect of 3-IAld-MP on parameters of liver damage by measuring hepatic-related enzymes, such as alkaline phosphatase (ALP), alanine aminotransferase (ALT), and total bilirubin [28]. As expected, DDC administration increased the serum levels of ALT and ALP in the active phase, with the levels remaining significantly higher in the recovery phase. 3-IAld-MP prevented ALT release, more than ALP, in the active DDC phase, and the levels remained low in the recovery phase (Figure 2B), demonstrating a protective effect of 3-IAld-MP on hepatocellular injury. Accordingly, the total serum bilirubin levels were decreased upon 3-IAld-treatment (Figure 2B). 3-IAld-MP also slightly reduced the porphyrin plugs in the liver (Figure 2C,D) as well as the production of inflammatory cytokines, such as IL-6, IL-17A, INF-γ, and, partially, TNF-α, (Figure 2E), a finding suggesting that preventing DDC uptake and restraining inflammation are both likely mechanisms underlying the efficacy of 3-IAld in murine sclerosing cholangitis. As a matter of fact, 3-IAld-MP administration greatly reduced the presence of inflammatory CD11b^+^ and F4/80^+^ cells in the liver (Figure 2F). Since liver chronic inflammation drives hepatic fibrosis, which is one pivotal feature in sclerosing cholangitis [26], we assessed fibrotic markers in the DDC-fed mice and the effects of 3-IAld-MP administration on those markers. Interestingly, 3-IAld-MP strongly reduced liver fibrogenesis in DDC-fed mice, as evaluated by alpha-smooth muscle actin (α-SMA) staining, a marker of hepatic stellate cells that mainly produce collagen in liver fibrogenesis [29], collagen fiber formation (Figure 3A,B), as well as pro-fibrotic cytokine levels, such as TGF-β and IL-9 (Figure 3C,D), both known to have a pivotal role in liver fibrogenesis [30,31]. Altogether, these data demonstrate that 3-IAld-MP prevents liver fibrosis in sclerosing cholangitis by modulating chronic inflammation in the liver. 

### 3.3. 3-IAld Prevents Liver Injury by Reducing DDC-Induced Intestinal Inflammation

**Figure 4:**
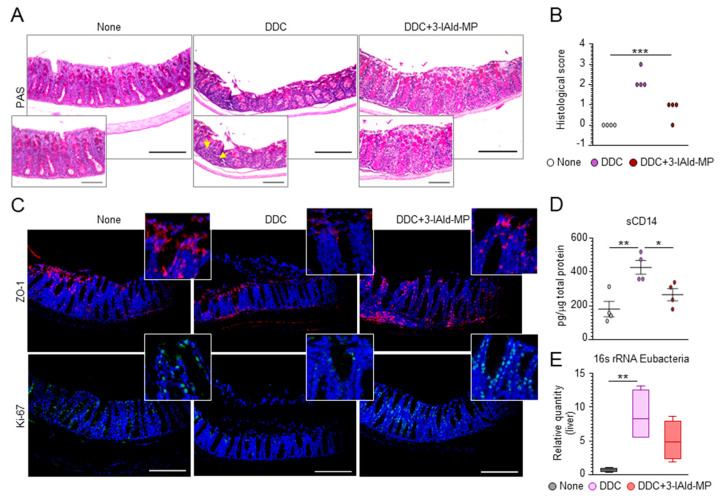
3-IAld-MP prevents liver injury by reducing DDC-induced intestinal inflammation. C57BL/6 mice were fed for 14 days with a 0.1% DDC-containing diet and subjected to a recovery time of 14 days. The mice were treated intragastrically with 3-IAld-MP every other day throughout the experiment. The mice were evaluated for (**A**) colon histology by PAS staining, (**B**) histological score, (**C**) ZO-1 and Ki-67 immunofluorescence, (**D**) sCD14 release in serum, and (**E**) Eubacteria expression in the liver by RT-PCR. Photographs were taken with a high-resolution microscope (Olympus BX51), 20× magnification (scale bars, 200 μm). Yellow arrows indicate inflammatory cells. Hoechst was used for counterstaining nuclei. The data are presented as the mean ± SEM and are representative of three experiments (*n* = 4 mice/group). * *p* < 0.05, ** *p* < 0.01, *** *p* < 0.001, treated vs untreated (none) mice, and DDC vs. DDC + 3-IAld. One-way ANOVA, Bonferroni post-test, (**B**) Kruskal–Wallis test.

The close relationship between PSC and IBD led us to postulate that reinforcing the epithelial barrier function and preventing microbial dysbiosis could be of therapeutic utility. Given that 3-IAld-MP enhances the intestinal mucosal barrier function through a variety of mechanisms [15], we asked whether its protective effect on liver injury may involve activity on the gut. As expected, and consistent with the observation that primary liver injury and cholestasis are associated with intestinal mucosal hypoplasia [28], DDC-induced liver damage was associated with alterations in colon morphology (Figure 4A,B) and a reduced expression of the tight junction zonula occludens 1 (ZO-1) and the proliferation marker Ki-67 (Figure 4C). 3-IAld-MP treatment promptly restored the intestinal mucosal and barrier and the epithelial permeability, as revealed by the amelioration of colon histopathology, the restoration of ZO-1 and Ki-67 expression (Figure 4A–C), and the decreased levels of soluble CD14 (sCD14) (Figure 4D), a marker of gut permeability [32]. The maintenance of intestinal paracellular integrity is critical to avoid bacterial translocation into the enterohepatic circulation and the ensuing liver inflammation. We found a lower abundance of total bacteria in the liver of 3-IAld-MP-treated mice compared to the untreated group (Figure 4E). These results indicate that 3-IAld-MP can act on the “gut–liver axis” by maintaining intestinal paracellular integrity, preventing bacterial translocation, and protecting from DDC-induced liver damage.

### 3.4. 3-IAld Prevents DDC-Liver Injury through the Microbiota-AhR-IL-22 Axis

**Figure 5:**
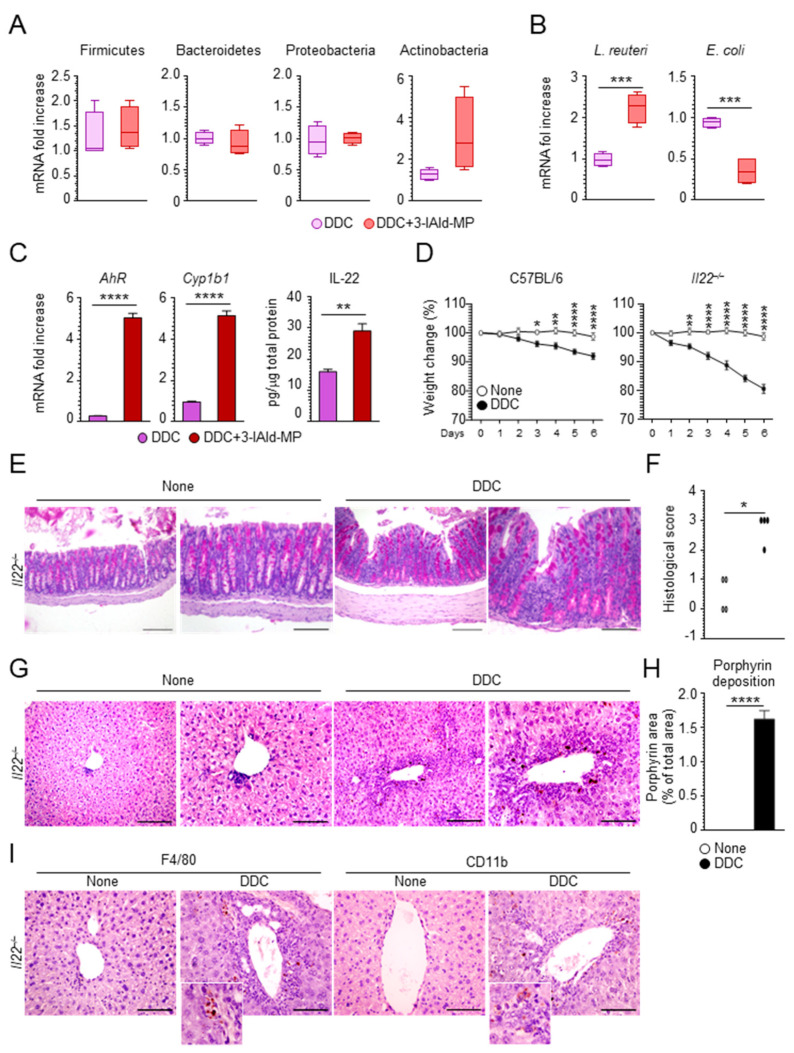
3-IAld-MP protects from sclerosing cholangitis through the microbiota-AhR-IL-22 axis. C57BL/6 mice were fed a 0.1% DDC-containing diet and subjected to a recovery time of 14 days. The mice were treated intragastrically with 3-IAld-MP every other day throughout the experiment and evaluated for (**A**) bacterial phyla expression, (**B**) *L. reuteri* and *E. coli* in fecal samples, (**C**) *AhR* and *Cyp1b1* expression by RT-PCR, and IL-22 production by ELISA in colon homogenates. *Il22^–/–^* mice were fed with a 0.1% DDC-containing diet for 6 days and evaluated for (**D**) % weight change, (**E**) colon histology by PAS staining, (**F**) histological score, (**G**) liver histology by H&E, (**H**) porphyrin deposition, and **(I)** CD11b^+^ and F4/80^+^ cells by immunohistochemistry. Photographs were taken with a high-resolution microscope (Olympus BX51), 20× and 40× magnification (scale bars, 200 μm and 100 μm). The data are presented as the mean ± SEM and are representative of three experiments (*n* = 4 mice/group). * *p* < 0.05, ** *p* < 0.01, *** *p* < 0.001, **** *p* < 0.0001, treated vs untreated (none) mice or DDC vs DDC + 3-IAld-MP. One-way ANOVA, Bonferroni post-test, two-tailed Student’s t-test, (**F**) Kruskal–Wallis test.

Gut microbial dysbiosis in patients with PSC is associated with hepatic inflammation [33]. Several studies have highlighted a specific gut microbiota composition that distinguishes PSC-gut inflammation from other IBD phenotypes [34,35]. To evaluate the effect of 3-IAld-MP in shaping the microbial composition in mice subjected to a DDC diet, we quantified indigenous bacteria by performing 16S ribosomal RNA sequence analysis in fecal samples. Although 3-IAld-MP did not affect the expansion of bacteria at the phylum level (Figure 5A), it sharply decreased the expansion of *E. coli* (Figure 5B), known to be implicated, together with *Klebsiella pneumoniae*, in liver pathologies, including PSC [1,36]. Of interest, 3-IAld-MP increased the expansion of *L. reuteri* (Figure 5B), which has been associated with protection from bile duct ligation-induced liver injury and fibrosis [37]. Consistent with the ability of *L. reuteri* to activate AhR-dependent functions [12], 3-IAld-MP activated AhR and promoted IL-22 production (Figure 5C). Considering the increased susceptibility of *Il22^–/–^* mice to the DDC toxicity treatment, as shown by the body weight loss (Figure 5D), colon (Figure 5E,F), and liver (Figure 5G,H) histopathology worsening, and inflammatory cells recruitment (Figure 5I), these data demonstrate that the improvement of liver damage and fibrosis upon 3-IAld-MP treatment may involve the microbiota-AhR-IL-22 axis.

## 4. Discussion

The close link between PSC and IBD points to the "gut–liver" axis as a key player in the pathogenesis of PSC and suggests that the modulation of this axis, including the microbiota, may offer opportunities for future interventions [7,8]. This study shows that the endogenous metabolite, 3-IAld, ameliorated the inflammatory pathology in murine PSC through a primary action on gut mucosal and microbial homeostasis. As such, it provides a proof of concept demonstration of the drugability of the gut–liver axis in PSC.

The AhR-IL-22 axis drives the maintenance of mucosal immune homeostasis in the gut [13] and the liver [17,18,20,21], where the prevention of fibrogenesis occurs through the inhibition of hepatic stellate cell activity, which includes the production of pro-fibrogenic cytokines, including TGF-β [38]. We found it of interest that 3-IAld greatly inhibited the production of TGF-β and IL9, along with the reduction of collagen deposition in the DDC-treated liver, a finding indicative of possible local activity of 3-IAld. Although not directly proved in this study, a recent pharmacokinetic study has revealed that 3-IAld is detectable in the liver of mice receiving 3-IAld-MP [14]. Moreover, we have evidence that 3-IAld activates AhR-dependent genes in a human hepatoma cell line in vitro (data not shown). Thus, local activity of 3-IAld in the liver is a plausible working hypothesis. 

However, in addition to likely local activity in the liver, our study clearly shows that 3-IAld activates the AhR-IL-22 axis in the gut, as already described [12,14,39,40]. The administration of 3-IAld potentiates the epithelial structure by increasing the expression of ZO-1 and Ki-67 and decreasing the levels of sCD14, thus favoring the restoration of the gut barrier function. As the integrity of the intestinal barrier is crucial for the maintenance of liver homeostasis, and a mutual relationship between the liver and the gut prevents potentially harmful substances translocating across the intestinal barrier [41], our results further strengthen the therapeutic value of 3-IAld in restoring the gut–liver axis functionality.

Indole metabolites produced by intestinal bacteria are known to control liver disease manifestation [42,43] through a variety of mechanisms in addition to AhR activation and IL-22 production [42]. We found that one additional mechanism through which 3-IAld may be beneficial at the gut–liver axis level involves the microbiota. Alterations in the intestinal microbiota play a role in the pathogenesis and progression of many disorders, including liver and gastrointestinal diseases [44]. Both qualitative (composition) and quantitative (amount) changes in gut microbes are associated with increased susceptibility to liver diseases [45]. We found that treatment with 3-IAld-MP significantly decreased the abundance of pro-inflammatory Enterobacteria, such as *E. coli*, while increasing the abundance of *L. reuteri*. Preliminary as they are, these data suggest an activity of 3-IAld on microbial cells and are consistent with the ability of indoles to act as intercellular signals in microbial communities [46].

In conclusion, this study provides further evidence of the beneficial effect microbial metabolites may have on the host pathophysiology. Safer than probiotics, microbial metabolites are receiving increasing attention for their pleiotropic effects, which include immunomodulatory, anti-inflammatory, antioxidant, and anti-cancer properties [47,48]. Microbial metabolites have drawn attention because of their clear chemical structure, safety dose parameters, long shelf life, and their ability to rescue gut health while preserving microbiota integrity [48]. The recently proposed “epithelial barrier hypothesis” points to the increase in epithelial barrier-damaging agents linked to industrialization, urbanization, and modern life as causative factors of the rise in allergic, autoimmune, and other chronic conditions [49]. This may broaden the therapeutic potential of microbial metabolites such as 3-IAld, and demands for drug delivery platforms for targeted therapies of a variety of clinical settings. For 3-IAld, in particular, its therapeutic activity in mice with cystic fibrosis [50] likely predicts an effect on extrapulmonary manifestations observed in these patients [51], such as cholangiopathy, to which, in addition to the genetic mutation, abnormal intestinal permeability combined with diet-induced dysbiosis are known to contribute [52,53].

## Data Availability

The data presented in this study are available on request from the corresponding author.
